# The Neonatal Intensive Care Unit: Environmental Stressors and Supports

**DOI:** 10.3390/ijerph15010060

**Published:** 2018-01-03

**Authors:** Kristen G. Williams, Kayla T. Patel, Julie M. Stausmire, Christy Bridges, Mary W. Mathis, Jennifer L. Barkin

**Affiliations:** 1 School of Medicine, Mercer University, 1550 College Street, Macon, GA 31207, USA; Kristen.Gayle.Williams@live.mercer.edu (K.G.W.); Kayla.Patel@live.mercer.edu (K.T.P.); bridges_cc@mercer.edu (C.B.); 2 Mercy St. Vincent Medical Center, Academic Research Coordinator, 2222 Cherry St. Suite 1300 MOB 2, Toledo, OH 43608, USA; 3 College of Health Professions, Mercer University, 3001 Mercer University Drive, Atlanta, GA 31404, USA; MATHIS_MW@mercer.edu

**Keywords:** NICU, NICU environment, maternal mental health, maternal functioning

## Abstract

The relationship between maternal mental health and infant development has been established in the literature. The Neonatal Intensive Care Unit (NICU) is a particularly challenging environment for new mothers as several natural processes are disrupted. The objective of this study is to elucidate protective factors and environmental deficits associated with the NICU. The experiences of forty-six (*n* = 46) mothers of infants admitted to a Level III NICU in the Midwestern United States, who responded to a related open-ended question, were analyzed thematically. Five themes related to the NICU environment emerged as being either stressful or helpful: (1) amount and quality of communication with medical staff, (2) bedside manner of medical staff, (3) feeling alienated from infant’s care, (4) support from other NICU mothers and families, and (5) NICU Physical Environment and Regulations. There is a need for medical staff training on awareness, communication, empathy, and other behaviors that might improve maternal (and parental) experiences in the NICU. The physical environment, including rules and regulations of the NICU, should be reexamined with family comfort in mind in addition to the clinical care of the infant.

## 1. Introduction

Giving birth to an ill or high-risk infant that requires admission to the Neonatal Intensive Care Unit (NICU) creates additional layers of responsibility for mothers who are already facing a major life adjustment [1,2]. This often portends to unforeseen complexities in daily life while both the child is in the NICU and after discharge given potentially complex home care plans. Challenges such as transportation to and from the NICU, visitation hours and balancing other aspects of family life can create a great deal of stress on parents of infants in the NICU [3]. These challenges can make it difficult for new parents to develop feeding schedules and establish secure bonding with their infant via quality time and skin-to-skin contact [3].

The NICU environment significantly impacts families [4] and mothers, in particular, who often assume the role of the primary caregiver [5]. Nearly 7% of births in the U.S. result in infants who require some degree of care in the NICU [6]. Anxiety, depression and posttraumatic stress disorder (PTSD) are common amongst parents with medically fragile infants in the NICU [4]. Holditch et al. [7] reported that 15% of mothers and 8% of fathers with premature infants admitted to the NICU experience postpartum PTSD. Additionally, between 39–63% of mothers with NICU infants experience post-partum depression (PPD) [4,8] compared to a PPD prevalence rate of 13–19% in their counterparts who do not have a baby in the NICU [9,10].

The impressive relationship between maternal mental health and infant development in the perinatal period has been discussed in the literature [9]. A 2013 study including 32 extremely premature neonates (<27 weeks) and their mothers examined factors that influence the amount of time that mothers visited with their infants in the NICU and participated in skin-to-skin care [11], a practice that has been strongly indicated in improved infant outcomes, particularly for premature neonates [12]. The results of the study indicated that both maternal stress in the NICU as well as maternal perceptions of how well the staff communicated with them played a significant role in visitation time and skin-to-skin care with their neonates [11]. Other factors such as maternal depression and anxiety have been linked to lowered capacity for decision-making and infant care as well as poor physiological and psychological outcomes for infants that may last through adolescence [12]. The relationship between maternal mental health and infant well-being further highlights the importance of identifying risk and protective factors for mothers whose infants are admitted to the NICU.

Common stressors that parents experience while having an infant in the NICU include anxiety about their infant’s well-being, struggling to meet the demands of parenting other children, loss of parenting role to NICU medical team, transportation barriers, and financial strain [13,14,15]. Due to the impact of these stressors and subsequent maternal adjustment on both maternal and infant health, NICU experiences are becoming critical area of study. A qualitative study by Heydarpour et al. [16] analyzed common factors that influence maternal adjustment amongst 17 mothers with babies in the NICU and found that common themes such as feelings of self-efficacy, social support, emotional turmoil, feelings of alienation, and interaction with healthcare providers played a critical role in adjustment to new motherhood in a NICU environment. A meta-analysis of 12 qualitative studies about maternal NICU experiences in mothers of pre-term infants resulted in 5 primary themes. These themes included challenges and anxiety surrounding the mother’s relationship with their newborn, challenges with establishing a sense of identity as a “normal” mother in a NICU environment, coping with a turbulent neonatal environment, striving to reclaim a care-giving role in the NICU, and relationships and communication with nurses [17].

The purpose of this particular investigation is to explore, through thematic analysis, the experiences of mothers of infants admitted to a Level III NICU in the Midwestern United States, who responded to an open-ended question focused on elucidating supportive and stressful features of the NICU environment. Level III NICUs are certified to provide the highest level of care available for critically ill newborn infants of any gestational age; they are equipped to deal with a wide range of complexity and risk related to the infant’s condition. Neonatologists, neonatal nurses, and respiratory therapists are on site 24/7. NICU parents are an inherently vulnerable population due to complicating circumstances. Additionally, our exploration of the literature indicates that significant work remains in terms of improving the NICU experience for families. A specific focus on environment is intended to identify deficits and protective features inherent to the setting. Ideally, results from this study and other related studies, would form the basis for evidence-based modifications to the NICU environment.

## 2. Materials and Methods

### 2.1. Setting and Recruitment

The current investigation is a secondary analysis from a parent study that included 146 participants. The study took place in a Level III Neonatal Intensive Care Unit (NICU) setting in the Midwestern United States between February 2015 and June 2016. The rooms within the NICU are set up in pods with four infants per pod; there are also four private rooms but the rest of the unit is open, with the pods sectioned off from a main hallway. Nurses are staffed according to critical status. For example, one infant may have two or more staff working with them. However, one nurse can potentially handle several infants at once who are stable and/or approaching discharge. While there are reclining chairs in each pod for a parent to rest, there are no overnight sleeping sites in the NICU itself. There is a “Home Away From Home” on site, which is similar to a Ronald McDonald House. That is, it is set up like a hotel with free kitchen and laundry areas, private rooms, and reserved parking.

**Figure 1:**
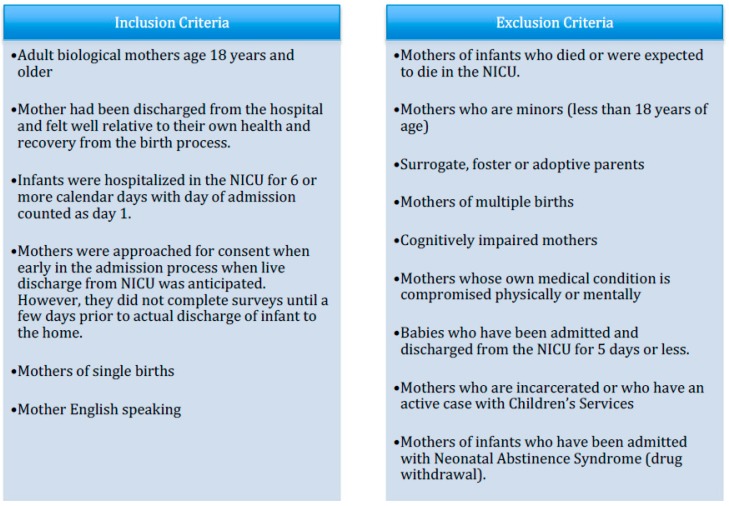
Inclusion/exclusion criteria of participants. NICU: Neonatal Intensive Care Unit.

Participants included mothers who had been discharged from the hospital following childbirth and whose infants were expected to survive but required NICU admission due to health complications following birth. The criteria used for the inclusion and exclusion of participants in the parent study can be found in Figure 1; methodological details such as inclusion criteria, consent procedures, and scope of data collection will also appear in other related publications. Both the Adult and Pediatric Institutional Review Boards of the affected institution approved this study (IRB # 1114103).

### 2.2. Procedure

An investigator approached potential subjects after the mother had been discharged from the hospital and the admitted infant was stabilized and generally expected to survive. Informed consent was obtained prior to enrollment. Separate signatures were obtained from the mother for the consent of herself and the minor child since both were subjects of the parent study. Enrolled participants were asked by a clinical study investigator to complete an assessment battery comprised of: (1) a sociodemographic survey, (2) the Barkin Index of Maternal Functioning-Neonatal Intensive Care Unit (BIMF-NICU) [1,18] the Edinburgh Postnatal Depression Scale (EPDS) [19], and (3) the Parental Stressor Scale: neonatal intensive care unit (PSS:NICU) [8]. The investigators were formally granted permission from the developer, Dr. Margaret Miles, to use the PSS:NICU. Participants completed the assessment approximately three days prior to the date that their infant was expected to be discharged from the NICU. Those who completed the study were compensated with two baby sleepers and a developmental toy.

### 2.3. Participants

Of 146 women, 46 responded to the final question on the PSS:NICU, inviting comments. The original wording from the PSS:NICU was “Feel free to write about other situations that you found stressful during the time that your baby was in the neonatal intensive care unit.” For study purposes the statement was modified to “You are welcome to write any comments about other situations that you found stressful or what was helpful to you during the time that your baby was in the neonatal intensive care unit.” This change was intended to elicit comments on what subjects found helpful as well as stressful. The information provided by these 46 women is the focus of the present investigation.

Of the 46 mothers who responded and are therefore included in this analysis, the mean age was 28.9 years (SD = 5.6) and they were predominately Caucasian (60%) and Non-Hispanic (95.6%). Most women had completed high school (97.8%) and were employed either full-time (46.7%) or part-time (24.4%). Less than half of the women had a college or postgraduate degree (33.3%) and slightly over half were married (51.1%). The mean EPDS score was 7.7 (SD = 4.3) and the average BIMF score was 96.1 (14).

This average EPDS score of 7.7 is less than the commonly used EPDS thresholds of 10 and 13 which suggest that follow-up is indicated [20]. While no established clinical threshold currently exists for the BIMF or the BIMF-NICU, a mean of 104 (SD = 11.8) was reported in study of low-income Ob/Gyn patients (*n* = 128) [21]. Further, in a small qualitative study (*n* = 24), again featuring low-income Ob/Gyn patients, an average BIMF score of 100.6 (SD = 10.8) was reported [22]. However, in studies including only women who were screen-positive for depression, the average BIMF score was 80 [18,23]. Table 1 describes the sociodemographic characteristics of the participants in further detail.

### 2.4. Data Analysis

All participant responses to the aforementioned study question were extracted and placed into a separate text file for the purposes of thematic analysis. The study analyst and the principal investigator, both trained in qualitative methods, reviewed the comments several times. Each developed a set of codes/themes to characterize the data. Discrepancies between the two sets of themes were related to naming/title rather than the underlying content/construct. The names/titles for the themes were rectified between the analysts and comments were subsequently grouped by theme. As a result, five primary (and mutually agreed upon) themes emerged.

## 3. Results

The five emergent themes were: (1) amount and quality of communication with medical staff, (2) bedside manner of medical staff, (3) feeling alienated from infant’s care, (4) support from other NICU mothers and families, and (5) NICU environment and rules. Participant quotations are presented to elucidate each theme. Quotations may appear more than once if applicable to multiple themes.

### 3.1. Amount and Quality of Communication with Medical Staff

Women often noted lack of quality communication with the nursing staff as a major stressor in the NICU:

Participant: *I found it extremely stressful that I never really knew what was going on. Aside from a few nurses, most of the time when I asked a question about why my baby was doing something (For example the “squeaky breathing”) the nurses just said it was normal. However, most of them did not take the time to explain why he was doing such things or what was happening.*

Participant: *During 2 procedures no one was able to give us an update—one was told to last and hour and 3.5 h later they were finished—we asked other nurses to check for us but they were only told their (the procedure) aren’t (wasn’t) done. When he has (was) admitted no one told us why other than his blood work was really bad, and 4 h later we were finally able to come down to (the) NICU. Someone could have explained more why he was rushed down from well-baby. We were very fearful and anxious which I believe could have been avoided with a little more communication.*

Conversely, good communication with NICU medical staff was often reported to have had a positive impact on the women’s experience in the NICU:

Participant: *What was helpful to me during our time here was the helpful nursing staff and great doctors. They were great at explaining things and it was reassuring to know that my baby was getting the best care possible. Rounding is a great thing.*

Participant: *Having the nurses sit down and explain what was truly going on with our baby helped (to) comfort us and lessen the stress we had.*

### 3.2. Bedside Manner of Medical Stafff

In the current study, bedside manner was defined as any nuances in body language, behavior and communication exhibited by medical staff during interactions with patients and their families that impacted patient satisfaction. Comments related to the bedside manner of the medical staff often included statements about the attitude, professionalism, and manner of care expressed by the nurses. Poor bedside manner was noted as a stressor. Examples of this manner as reported by participants included the following:

Participant: *… I was reprimanded rudely when I came back from lunch to find my baby had spit up on herself and pooped her diaper and I changed her and cleaned her up in between her feeding time… NOT OK. Some RN’s are quite rude if you’re not getting enough breast milk/don’t have enough.*

Participant: *The most frustrating thing is when nurses don’t try. For example just hook the baby to the feeding tube so they can sit and talk on the phone (yes, personal phone calls).*

Participant: *I love most of the nurses but there are a few that I have seen to be careless and too rough with the babies. This made me highly upset because they were so careless and rough. That is unacceptable—these babies are tough but they go through too much and are too precious to be treated that way.*

Other women expressed positive comments about the bedside manner of the medical staff noting the positive impact that it had on their experience in the NICU:

Participant: *The nursing staff however was very helpful in assisting and informing me about breastfeeding. (Names specific nurse) was my favorite! She was so helpful and kind. I would describe her as compassionate and yet professional.*

Participant: *I really appreciated the nurses giving me my space but still checking in on me from time to time. They were also very helpful with supplies for breastfeeding. They never seem to get irritated or frustrated which makes me as a parent feel more comfortable and at home.*

### 3.3. Feeling Alienated from Infant’s Care

Some women expressed that they felt excluded from the process of caring for their infant or that they felt “unwelcomed” in the NICU:

Participant: *It also seemed like the nurses would rather do things themselves than have me do it (i.e., feedings, baths). It seems like we are in the way and that’s frustrating.*

Participant: *I was a little disappointed I did not get to witness my baby’s first bath. I didn’t know when they were planning to bathe her.*

Participant: *… Her nurse came by and told me that it wasn’t her care hour and it would be best if I was there then instead. It made me feel completely unwelcome and as if I wasn’t allowed to see my own child. I had a meltdown. I went back to my room and sobbed.*

### 3.4. Support from Other NICU Moms and Families

Communicating with other mothers in the NICU was a factor that reportedly helped to alleviate some of the stresses of having an infant in the NICU. Several women found comfort and support in their shared experiences:

Participant: *Staying at hospital-based housing (and) talking to other families that have had their babies early is nice.*

Participant: *Just hearing stories from other women that went through the same thing and their child is/was grown or older and going great, gives me hope and praying makes everything better.*

Participant: *What was helpful to me during our time here was … talking with other moms going through similar situations and being able to empathize and talk with them.*

### 3.5. NICU Physical Environment and Regulations

Numerous participants described the NICU environment as either a stressor or highlight of their experience. This included things such as the physical environment of the NICU as well as the rules and regulations that were enforced there:

Participant: *Having to choose between family members to only allow for 3 at a time, lack of waiting area for visitors waiting their turn. Having to coordinate feeding times with the cafeteria hours, not eating due to cafeteria being closed after feedings. Put vending options by unit. Bathrooms available in unit!!! Very hard after surgery to walk far to use bathroom when the one bathroom for the unit is being used.*

Participant: *Being able to call/visit 24/7 helped. Not being allowed to sleep here was stressful. Poor support trying to pump/breastfeed was stressful. Having rotating doctors was comforting because they all have different views about the situations and medical conditions.*

Participant: *I feel it is not fair that every baby (is) not in a room with their parents. And (parents) not (being) able to stay, (but) wanting to be with their premie child—something needs to change with that. Because (for) parents who (have) never been through things like this, which is the NICU, (it) is not fair to them. It’s already (missing word here) that we parents (are) not able to take them home. To know you can’t stay the night—I don’t like. How is that you can visit 24 h but not sleep? It’s very stressful on parents.*

## 4. Discussion

The aim of the current study was to conduct an analysis of qualitative data in order identify prominent environmental stressors and supports of the NICU. Results were analyzed and five themes specific to environment emerged. These themes included Amount and Quality of Communication with Medical Staff, Bedside Manner of Medical Staff, Feeling Alienated from Infant’s Care, Support from Other NICU Moms and Families, and NICU Physical Environment and Regulations. The findings in this investigation confirm and supplement some of the findings from previous (and related) studies. They add to the growing body of evidence that highlights the significance of the NICU environment as it relates to family health.

### 4.1. Amount and Quality of Communication with Medical Staff

Communication was a key theme throughout the literature; therefore it was foreseeable that communication with medical staff would be identified by mothers as significant experience in the NICU. Several studies addressing parental satisfaction in the NICU have corroborated this finding. In their meta-synthesis of 14 studies on maternal NICU experiences, Aagard et al. [17] identified a theme that they called “Mother-nurse relationship: Continuously answering questions through chatting and sharing of knowledge.” This theme refers to the importance of communication between nurses and mothers. This dynamic can be helpful when working optimally and but contributes to stress otherwise.

In the current study, communication was often at the core of the mother’s experiences in the NICU whether positive or negative, even if the word “communication” was not specifically used in the participant’s response. While Quality and Amount of Communication was listed as its own separate theme, there was a great deal of overlap between this theme and other themes such as Bedside Manner, Feeling Alienated from Infant Care, and NICU Environment and Rules. This is likely because quality and amount of communication between mothers and medical staff is linked to numerous outcomes. For example, one mother expressed disappointment in missing her baby’s first bath because the bath time had not been communicated to her. This statement was listed under both the alienation theme and the communication theme because the alienating experience could potentially have been prevented via better communication between the nursing staff and the mother about the newborn’s expected bath time. Similar trends exist within the Bedside Manner theme as well as the NICU Environment and Rules theme where communication was a contributing factor to a positive or negative experience.

Weiss et al. [24] conducted a study examining provider communication interventions in the NICU and their effects on patient satisfaction. One of these interventions included brief education for providers about communication with family members of NICU patients. Another intervention included providing patients with contact cards so that they had access to provider’s names, job descriptions and contact information. The final intervention included displaying large posters at the entrance of the NICU that included provider names, pictures and titles. The investigators found that these interventions that helped providers to increase their availability for and communication with parents led to greater parental satisfaction [24]. Interventions such as those employed in this study could prove beneficial for decreasing maternal stress in the NICU environment. With communication being a common thread throughout, it is clear that it must become a focus of staff education and training.

### 4.2. Bedside Manner of Medical Staff

Bedside manner is a difficult concept to classify due to its somewhat vague nature and definition. Merriam Webster’s Dictionary defines bedside manner as “the manner that a physician assumes toward patients [25]”. In the current study, bedside manner was broadly defined as any nuances in body language, behavior and communication exhibited by medical staff during interactions with patients and their families that impacted patient satisfaction. Due to this comprehensive definition, there is great diversity in the experiences cited within the bedside manner theme.

Some women commented on the attentiveness and patience of the nurses, or lack thereof. Others commented on how helpful it was when doctors rounded frequently on their infants and answered questions. Numerous other mothers made comments about how stressful it was when nurses had negative attitudes or when they perceived nurses as being physically rough with the babies during care and procedures.

Person et al. [26] reviewed the literature on bedside manner and conducted a concept analysis of behaviors regarded as positive or negative by patients on a consistent basis in order to gain a deeper understanding of the bedside manner concept. The themes generated by their analysis included positive behaviors, such as displays of respect, courtesy, and listening as well as negative behaviors, such as arrogance, indifference and disrespect [26]. Nuances such as these were also seen under the bedside manner theme in the current study. One mother referred to some of the nurses as “snobby” and felt that they were unable to empathize with her. Another woman implied that one nurses’ lack of empathy contributed to her negative bedside manner.

Statements such as these highlight the continued need for more extensive training on awareness, warmth and empathy amongst nursing staff in order to facilitate a less stressful and more therapeutically effective environment for mothers and their medically fragile infants in the NICU. Roter [27] reviewed studies about the patient-physician relationship and identified multiple nuances, particularly in communication, that were linked to more positive patient outcomes. Physician displays of empathy, responsiveness to patient emotions, and inclusion of patients by asking questions and encouraging patients to ask questions as well often led to more positive health outcomes and decreased psychological distress amongst patients [27].

While bedside manner continues to be a broad term that encompasses many things, the results of this and previous literature imply that “bedside manner” is complex and comprised of the detailed interactions that medical staff have with patients. Future research should continue to parse out the intricacies of this term so that clinical interventions can be tailored specifically to the nuances that are most important to patients. Provider awareness should be a key focus of clinical intervention.

### 4.3. Feeling Alienated from Infant’s Care

It is not surprising that women expressed feelings of alienation and exclusion from their infant’s care. Heydarpour et al. [16] refer to a similar theme that they termed “feelings of alienation” denoting the uncertainty and lack of preparedness mothers felt with regard to caring for their premature infant in a NICU environment. In their meta-synthesis of 14 studies, Aagard et al. [17] identified a theme titled, “challenges with establishing a sense of identity as a ‘normal’ mother in a NICU environment” that encompass similar concepts to alienation. Mothers in the current study expressed a similar sentiment of feeling like a foreigner in the NICU environment and, more specifically, feeling excluded from their infant’s care routine by the trained medical staff.

These feelings of alienation are likely the combined result of the time demands on the NICU’s clinical staff as well as lack of communication between staff and mothers about infant care and how mothers can actively participate. Regardless of the reason, standard models do not seem equipped to integrate parents directly into the infant-care process in the NICU. Some mothers reported disappointment about missing out on activities, such as bath time, while other mothers reported feeling generally unwelcomed or as if they were in the way of nurses and staff. Medical staff is often under a great deal of pressure due to large patient loads, time constrains and the life-or-death reality of the NICU; however these responses implicate a need for nurses to play a larger role in encouraging mothers to participate in the care of their infants as much as possible and providing them with concrete examples of how to do so.

### 4.4. Support from Other NICU Moms and Families

Social support has been identified as a protective factor for individuals, particularly in stressful situations such as parenting [28]. Mothers in the current study expressed the importance of social support, particularly support from other mothers and families who have or have had infants in the NICU. Heydarpour et al. [16] found that social support from other mothers had a positive effect on adaptation to the role of motherhood in mothers of preterm infants in the NICU.

Potential clinical implications of this finding may include programs that facilitate peer support for mothers and families of babies in the NICU. Preyde et al. [29] evaluated the effectiveness of a “buddy” program for mothers with preterm infants in the NICU. The objective of this program was to pair mothers with preterm NICU infants with other mothers who had experienced having a preterm infant in the NICU in the past. The experienced mothers provided mostly telephone support. Participants in the intervention group reported lower levels of anxiety and depression as well as higher levels of perceived social support [29]. Other studies that have examined support groups have also yielded positive results with participants in the intervention groups reporting less anxiety and scoring more positively on measures of self-esteem and parent-child interaction [30]. The information gleaned from the current study as well as the literature on effective clinical interventions underscores the need for such support programs to become lasting component of the NICU environment.

### 4.5. NICU Physical Environment and Regulations

The theme of NICU Physical Environment and Regulations encompasses everything from the physical characteristics of the NICU (i.e., medical machines and devices, alarms, hectic interactions with medical staff, distressed families, etc.) to the rules of the NICU and the manner in which they were enforced.

It became apparent through participant comments that thorough explanation of medical equipment, its purpose and the meanings behind alarms and alerts helped relieve maternal stress a great deal. Multiple mothers also commented about inconveniences such as bathroom location and made suggestions for improvement of the physical environment such as installing vending machines in the waiting areas. Stacey, Osborn and Salkovskis [31] conducted a study assessing factors that helped parents cope with the NICU and found that the physical environment of the NICU played a critical role in parental ease and satisfaction. The link between the physical NICU environment and parental wellbeing has strong indications for practical quality improvements that promote parental convenience and comfort.

In the current study, the most frequent comments within this theme were regarding rules in the NICU and the manner in which they were enforced by nursing staff. Multiple women complained about frustrating rules that interfered with their ability to care for or spend time with their babies, and more than one mother complained about nurses enforcing the rules in ways that they perceived to be rude or hurtful. One mother expressed frustration about rules not being uniformly enforced by nurses, leading to confusion amongst patients. 

Baird [32] conducted a study on the impact of Pediatric Intensive Care Unit (PICU) rules on patient and family-centered care PFCC. Through their qualitative analysis of interviews with five mothers and two fathers with children in the PICU as well as 12 PICU nurses, it was concluded that unit rules were a significant source of contention between parents and nurses. Parents felt particularly frustrated by the rules that prevented them from spending the desired amount of time with their children. The investigation illuminated a discrepancy between nurse training in rule enforcement and the updated and more progressive goals of PFCC. The investigators speculated that some of the PICU rules and the means by which they were enforced originated from an outdated school of thought that placed the convenience of medical staff above the convenience and needs of families because families were viewed as visitors as opposed to an integral part of the care team for their children [32]. While this study was specific to the PICU, many similarities exist between the needs and concerns of parents in this study and those of the mothers in the NICU study.

The results of the current study in concordance with the literature provide a strong basis for the need of NICU facilities to clarify their mission amongst medical staff and patients and revise their rules to assure that they are current, necessary and in concordance with the mission. This clarity in addition to quality nurse training regarding the mission and respectful and empathetic rule enforcement has the potential to reduce maternal stress levels and create a more positive environment in the NICU.

### 4.6. Strengths and Limitations

The use of an open-ended question to assess stressors and supports in the NICU is a strength of this study. This allowed participants to share their experiences without the constraints associated with closed-ended questions and Likert Scale response choices. The timing of the survey administration was also a strength of the study as women completed the survey at the end of their baby’s stay in the NICU, which allowed them time to form opinions and give feedback; the timing also likely mitigated potential recall bias as the experience was in progress and not several weeks previous. The relatively large sample size is a strength of this study as well. The current investigation included 46 participants. Sample sizes of less than 30 participants are more prevalent in qualitative literature. Sample diversity was strength of this investigation. There was diversity amongst the sample with regard to level of education. Slightly over half of the sample had some amount of college education that included an associates or technical degree (17.8%) or a college or postgraduate degree (33.3%) while the other half of the sample had a high school diploma or GED (46.7%). The sample was also racially diverse (60% White, 28.9% Black and 4.4% Hispanic), which has a positive impact on the generalizability of these results. Finally, study participants were primed for the open-ended question related to environment after having completed the PSS:NICU items.

One limiting factor of the open-ended question is that intriguing topics could not be probed further, as possible during focus groups or patient interviews. The participants could not be asked to expound further upon comments or to provide clarification about vague or confusing statements, which may have granted better insight for investigators. Self-selecting bias is also noted as a limitation of the current study. Only 46 of the total 146 total participants in the parent study answered the open-ended question for the current investigation. This has the potential to perpetuate bias if the women who responded to the question shared demographic, psychosocial or other qualities that influenced their likelihood to provide commentary. For example, it was noted that the ratio of Medicaid to private insurance amongst participants in the current investigation differed from that of the parent study (63% Medicaid, 37% Private) with more women in the current study being covered by private insurers (44.4% Medicaid, 55.6% Private). This may be indicative of discrepancies in expectation for medical care between the privately insured and those insured by Medicaid. Those who are privately insured may have a greater breadth of experience with different medical facilities giving them stronger opinions and making them more likely to provide feedback. This was a single site study, therefore patient feedback (and all data collection) was restricted to one geographic location in the United States. However, several of the themes are corroborated by other cited studies, which took place in a variety of locations.

Since mothers completed the survey at the end of their baby’s NICU stay, it should also be noted that length of infant’s NICU stay before discharge varied amongst participants. However, while length of stay varied, uniformly assessing women at infant discharge meant that all women had, at a minimum, several days experience in the NICU environment.

## 5. Conclusions

The link between maternal stress and infant health and development has been stressed in the literature [8]. According to Gonya et al. [11], women who exhibit higher levels of stress interact less with their infants in the NICU and are less likely to participate in skin-to-skin contact which has been proven to have beneficial effects on newborn health and recovery from complications [11]. This research as well as the results from the current study suggest a need for medical staff in the NICU to become more aware of the needs and feeling of the mothers and to make a viable effort to decrease maternal stress levels through communication and empathy, whenever possible.

One major implication of this study is that these themes are not mutually exclusive. Good bedside manner cannot be practiced without effective communication between patients and medical staff and effective communication is necessary for mothers to feel included and not alienated from their infant’s care. Future research should focus on parsing out specific factors encompassed by the term bedside manner as well as those nuances in communication that impact maternal stress in the NICU environment since many of these factors likely have a direct or indirect impact on other themes discussed in this study. NICU interventions that are successful at increasing communication, empathy, and awareness should be considered essential continuing education for NICU medical staff. This report indicates that much work remains related to improving the NICU environment for families.

### Clinical Implications

Clinical implications include the need for regular evidence-based training for medical staff on awareness, communication, empathy and other behaviors that might improve bedside manner and promote inclusion of mothers in infant care. Communication may also help with NICU rules and environment. Many mothers in the current study experienced frustration with NICU rules and did not always understand their relevance. Clearly communicating rules to mothers and explaining their purpose and importance in an empathetic and respectful manner may improve maternal satisfaction and reduce stress in the NICU. Revising outdated or unnecessary rules may also serve to reduce maternal stress. Results of the current investigation and previous literature also create a strong basis for implementation of in-house peer support programs for mothers of NICU infants in order to decrease maternal stress levels.

Practices may also want to consider models of Family Integrated Care (FIC) that are applicable to the NICU setting [33]. O’Brien et al. [33] describe a FIC model in which parents are considered an “integral part of the NICU team.” In this model, the nurses are mentors to the parents who are trained to provide all forms of infant care with the exception of intravenous fluid and medication administration. This type of model may well address parents’ concern of feeling alienated from infant care in the NICU setting. Parental self-care should be strongly encouraged by healthcare providers as self-neglect is a hazard of the postpartum period [34].

## Figures and Tables

**Table 1:** Characteristics of Study Population (*n* = 45).

Categorical Variables-Mother	*n*	%
Race		
White	27	60.0
Black	13	28.9
Other	5	11.1
Hispanic		
Yes	2	4.4
No	43	95.6
Marital Status		
Married	23	51.1
Not Married	22	48.9
Education		
<High School	1	2.2
High School diploma or GED	21	46.7
Associate degree/Technical degree	8	17.8
College degree/Postgrad	15	33.3
Insurance		
Medicaid	20	44.4
Other/Private	25	55.6
Employment Status		
Full-time	21	46.7
Part-time	11	24.4
Unemployed	13	28.9
Delivery Type		
Vaginal	15	33.3
C-section	30	66.7
Other children admitted to NICU		
Yes	4	9.1
No	40	90.9
**Continuous Variables-Mother**	**N**	**Mean (SD)**
Mother’s age (years)	45	28.9 (5.6)
Length of Stay (days)	45	4.1 (1.4)
Total number of adults in home	45	2.0 (0.7)
Total number of children	45	1.7 (1.0)
BIMF-NICU	45	96.1 (14)
EPDS	45	7.7 (4.3)
PSS:NICU	45	2.9 (0.9)

Abbreviations: EPDS = Edinburgh Postnatal Depression Scale; BIMF = Barkin Index of Maternal Functioning-Neonatal Intensive Care Unit; PSS:NICU = Parental Stress Scale: Neonatal Intensive Care Unit; Note: 46 participants provided comments for NICU stressor and supports; however, due to missing sociodemographic data this table reflects the data of only 45 participants.

## References

[1] Barkin J.L., Wisner K.L., Bromberger J.T., Beach S.R., Terry M.A., Wisniewski S.R. (2010). Development of the Barkin index of maternal functioning. J. Women’s Health.

[2] Barkin J.L., Wisner K.L., Bromberger J.T., Beach S.R., Wisniewski S.R. (2010). Assessment of functioning in new mothers. J. Women’s Health.

[3] Lee H.C., Martin-Anderson S., Dudley R.A. (2012). Clinician perspectives on barriers to and opportunities for skin-to-skin contact for premature infants in neonatal intensive care units. Breastfeed. Med..

[4] Del Fabbro A., Cain K. (2016). Infant Mental Health and Family Mental Health Issues. Newborn Infant Nurs. Rev..

[5] Pleck J.H., Masciadrelli B.P., Lamb M.E. (2004). Paternal involvement by U.S. residential fathers: Levels, sources, and consequences. The Role of the Father in Child Development.

[6] Harrison W., Goodman D. (2015). Epidemiologic trends in neonatal intensive care, 2007–2012. JAMA Pediatr..

[7] Holditch-Davis D., Bartlett T.R., Blickman A.L., Miles M.S. (2003). Posttraumatic stress symptoms in mothers of premature infants. J. Obstet. Gynecol. Neonatal Nurs..

[8] Miles M.S., Holditch D., Schwartz T.A., Sher M. (2007). Depressive symptoms in mothers of prematurely born infants. J. Dev. Behav. Pediatr..

[9] O’Hara M.W., McCabe J.E. (2013). Postpartum depression: Current status and future directions. Annu. Rev. Clin. Psychol..

[10] Feldman R., Granat A., Pariente C., Kanety H., Kuint J., Gilboa-Schechtman E. (2009). Maternal depression and anxiety across the postpartum year and infant social engagement, fear regulation, and stress reactivity. J. Am. Acad. Child Adolesc. Psychiatry.

[11] Gonya J., Nelin L.D. (2013). Factors associated with maternal visitation and participation in skin-to-skin care in an all referral level IIIc NICU. Acta Paediatr..

[12] Feldman R., Rosenthal Z., Eidelman A.I. (2014). Maternal-preterm skin-to-skin contact enhances child physiologic organization and cognitive control across the first 10 years of life. Biol. Psychiatry.

[13] Holditch-Davis D., Miles M.S. (2000). Mothers’ stories about their experiences in the neonatal intensive care unit. Neonatal Netw..

[14] Singer L.T., Fulton S., Kirchner H.L., Eisengart S., Lewis B., Short E., Min M.O., Kercsmar C., Baley J.E. (2007). Parenting very low birth weight children at school age: Maternal stress and coping. J. Pediatr..

[15] Hall S., Hynan N., Phillips R., Press J., Kenner C., Ryan D.J. (2015). Development of program standards for psychosocial support of parents of infants admitted to a neonatal intensive care unit: A national interdisciplinary consensus model. Newborn Infant Nurs. Rev..

[16] Heydarpour S., Keshavarz Z., Bakhtiari M. (2017). Factors affecting adaptation to the role of motherhood in mothers of preterm infants admitted to the neonatal intensive care unit: A qualitative study. J. Adv. Nurs..

[17] Aagaard H., Hall E.O. (2008). Mothers’ experiences of having a preterm infant in the neonatal care unit: A meta-synthesis. J. Pediatr. Nurs..

[18] Barkin J.L., Wisner K.L., Wisniewski S.R. (2014). The psychometric properties of the Barkin Index of Maternal Functioning. J. Obstet. Gynecol. Neonatal Nurs..

[19] Cox J.L., Holden J.M., Sagovsky R. (1987). Detection of Postnatal Depression: Development of the 10-item Edinburgh Postnatal Depression Scale. Br. J. Psychiatry.

[20] Wisner K.L., Sit D.K., McShea M.C., Rizzo D.M., Zoretich R.A., Hughes C.L., Eng H.F., Luther J.F., Wisniewski S.R., Costantino M.L. (2013). Onset timing, thoughts of self-harm, and diagnoses in postpartum women with screen-positive depression findings. JAMA Psychiatry.

[21] Barkin J.L., McKeever A., Lian B., Wisniewski S.R. (2017). Correlates of Postpartum Maternal Functioning in a Low-Income Obstetric Population. J. Am. Psychiatr. Nurses Assoc..

[22] Barkin J.L., Willis G.B., Hawkins K.C., Stanfill-Thomas T., Beals L., Bloch J.R. (2017). Semantic Assessment of the Barkin Index of Maternal Functioning in a Medically Underserved Obstetric Population. Perspect. Psychiatr. Care.

[23] Barkin J.L., Wisner K.L., Bromberger J.T., Beach S.R., Wisniewski S.R. (2016). Factors associated with postpartum maternal functioning in women with positive screens for depression. J. Women’s Health.

[24] Weiss S., Goldlust E., Vaucher Y.E. (2010). Improving parent satisfaction: An intervention to increase neonatal parent-provider communication. J. Perinatol..

[25] Merriam-Webster Online. https://www.merriam-webster.com/dictionary/bedside%20manner?utm_campaign=sd&utm_medium=serp&utm_source=jsonld.

[26] Person A., Finch L. (2008). Bedside Manner: Concept Analysis and Impact on Advanced Nursing Practice. Int. J. Adv. Nurs. Pract..

[27] Roter D. (2000). The enduring and evolving nature of the patient-physician relationship. Patient Educ. Couns..

[28] Barkin J.L., Bloch J.R., Hawkins K.C., Thomas T.S. (2014). Barriers to optimal social support in the postpartum period. J. Obstet. Gynecol. Neonatal Nurs..

[29] Preyde M., Ardal F. (2003). Effectiveness of a parent “buddy” program for mothers of very preterm infants in a neonatal intensive care unit. CMAJ.

[30] Roman L.A., Lindsay J.K., Boger R.P., DeWys M., Beaumont E.J., Jones A.S., Haas B. (1995). Parent-to-parent support initiated in the neonatal intensive care unit. Res. Nurs. Health.

[31] Stacey S., Osborn M., Salkovskis P. (2015). Life is a rollercoaster … What helps parents cope with the Neonatal Intensive Care Unit (NICU)?. J. Neonatal Nurs..

[32] Baird J., Davies B., Hinds P.S., Baggott C., Rehm R.S. (2015). What Impact Do Hospital and Unit-Based Rules Have Upon Patient and Family-Centered Care in the Pediatric Intensive Care Unit?. J. Pediatr. Nurs..

[33] O’Brien K., Bracht M., Macdonell K., McBride T., Robson K., O’Leary L., Christie K., Galarza M., Dicky T., Levin A. (2013). A pilot cohort analytic study of Family Integrated Care in a Canadian neonatal intensive care unit. BMC Pregnancy Childbirth.

[34] Barkin J.L., Wisner K.L. (2013). The role of maternal self-care in new motherhood. Midwifery.

